# The Dysfunction of the Cerebellum and Its Cerebellum-Reward-Sensorimotor Loops in Chronic Spontaneous Urticaria

**DOI:** 10.1007/s12311-018-0933-6

**Published:** 2018-03-24

**Authors:** Yuming Wang, Jiliang Fang, Ping Song, Yan Bao, Wenwen Song, Jiao Liu, Courtney Lang, Kristen Jorgenson, Minyoung Jung, Dong Shen, Shasha Li, Ruirui Sun, Xu Ding, Jiao Yang, Xiao Meng, Ning Wang, Zhifang Yan, Yuhe Yan, Qian Kong, Ying Dong, Fangyuan Cui, Yiheng Tu, Bingnan Cui, Jian Kong

**Affiliations:** 1 Department of Dermatology, Guang’anmen Hospital, China Academy of Chinese Medical Sciences, Beijing, 100053 China; 2 Department of Psychiatry, Massachusetts General Hospital, Harvard Medical School, Charlestown, MA 02129 USA; 3 Department of Radiology, Guang’anmen Hospital, China Academy of Chinese Medical Sciences, Beijing, 100053 China; 4 National-Local Joint Engineering Research Center of Rehabilitation Medicine Technology, Fujian University of Traditional Chinese Medicine, Fuzhou, Fujian 350122 China

**Keywords:** Resting-state functional magnetic resonance imaging (rs-fMRI), Itch, Chronic spontaneous urticaria, Cerebellum, Reward, Sensorimotor

## Abstract

Chronic spontaneous urticaria (CSU) is a common itchy skin disease. Despite its prevalence, the neuropathology of CSU is uncertain. In this study, we explored resting state functional connectivity (rs-FC) changes in CSU, as well as how the symptom changes following intervention can modulate rs-FC. Forty patients and 40 healthy controls (HCs) were recruited. Following an intervention, 32 patients participated in a second scan approximately 6 weeks after the first scan. Compared with healthy controls, CSU subjects exhibited higher regional homogeneity (ReHo) values in the cerebellum, which were positively associated with urticaria activity scores over 7 days (UAS7) at baseline. After an intervention accompanied with clinical improvement, we found that ReHo values decreased at the cerebellum and increased at the bilateral primary somatosensory cortex (SI)/primary motor cortex (MI)/supplementary motor area (SMA). Using the cerebellum as a seed, CSU subjects exhibited increased rs-FC with reward regions when compared with HCs and exhibited decreased rs-FC at the right orbitofrontal cortex and right sensorimotor region following the intervention. The improvement rate values were positively associated with reduced rs-FC values in the two regions. Using the cluster of SI/MI/SMA as a seed, CSU patients exhibited decreased rs-FC with the left putamen, caudate, accumbens, and thalamus following the intervention. These results demonstrate the altered cerebellar activity and cerebellum-reward-sensorimotor loops in CSU.

## Introduction

Chronic spontaneous urticaria (CSU) is a common disorder characterized by the recurrent (longer than 6 weeks) presence of spontaneous, transient, and itchy wheals [1]. CSU is a major burden on affected individuals and has the potential to severely impair individual’s health-related quality of life [2, 3]. Literature suggests that 0.5–1.0% of the population suffers from CSU at any given time [4]. Although it is recognized that the classical itchy pathway in CSU is histamine-mediated [5], there is a lack of understanding of the neuropathology of CSU and consequently, limited treatment options. Current treatment for CSU is far from satisfactory; standard therapy with regular doses of H1-antihistamines is ineffective for more than 50% of patients with CSU [4].

The brain is the regulating center of itch. Recently, brain-imaging tools have been used to explore the mechanism of itch, a key symptom of CSU, and have significantly enhanced our understanding of its central mechanism. These studies reveal that many regions may be involved in the brain processing of itch, including the primary somatosensory cortex (SI), secondary somatosensory cortex (SII), primary motor cortex (MI), premotor cortex (PM), supplementary motor area (SMA), prefrontal cortex (PFC), cingulate cortex, precuneus, striatum, thalamus, and cerebellum [6–10].

In recent decades, resting-state functional magnetic resonance imaging (rs-fMRI) has been applied to investigate the intrinsic functional organization of the brain [11–13]. There are many resting-state fMRI data analysis methods, some of which focus on long-range connectivity among different brain regions, such as seed-based resting state functional connectivity [14], and others that focus on local connectivity and examine the properties of spontaneous local brain activity, such as regional homogeneity (ReHo). ReHo is a method that can be used to characterize the synchronization of fluctuations of blood oxygen level-dependent (BOLD) signals among neighboring voxels within a single region, by which we can explore possible synchronization and coordination abnormalities of spontaneous neuronal activities in corresponding brain regions [15]. One characteristic of local connectivity methods, such as ReHo, is that unlike distant connectivity methods that explore the association between different brain regions or networks, local connectivity methods can help target key regions involved in the neuropathology of disorders [15, 16].

Combining both local and distant resting-state methods, we explored (1) the differences in resting state brain activity between CSU patients and matched healthy controls (HCs) and (2) how longitudinal treatment can modulate brain functional connectivity of CSU patients. To avoid the confounding factor of variation in itching intensity, all scans were applied while the CSU patients did not have itching. We hypothesized that (1) CSU patients would be associated with altered ReHo values in brain regions involved in chronic itch, including sensorimotor related regions, (2) altered ReHo regions would be normalized after effective treatment, and (3) The resting-state functional connectivity (rs-FC) of key regions associated with ReHo changes would also be associated with other circuit changes such as in the reward circuitry.

## Methods

The research protocol has been approved by the Institutional Ethics Committee of Guang’anmen Hospital affiliated with the China Academy of Chinese Medical Sciences. Informed consent was obtained from all participants.

### Subjects

#### CSU Patients

All patients were recruited from the Department of Dermatology at Guang’anmen Hospital. Patients with a documented history of CSU, characterized by transient, itchy wheals of unknown etiology, occurring regularly for 6 weeks or more prior to consent acquisition, were recruited.

Inclusion criteria were as follows: (1) ages 18 to 60 years, (2) a urticaria activity score over 7 days (UAS7) [17] > 14, (3) right handed, and (4) have a clear asymptomatic stage more than 3 h in the daytime without H1-antihistamine treatment.

Exclusion criteria were as follows: (1) use of sedating H1-antihistamines, corticosteroids, biologics, psychotropic drugs, or opioids in the past 3 months; (2) pain; (3) pregnant or lactating women; (4) current or history of psychiatric or neurological diseases, head trauma, or loss of consciousness; (5) claustrophobia; (6) metal implants; and (7) suffer from other skin diseases.

#### HCs

Right-handed HCs were gender and age matched with CSU patients.

### Intervention

All patients received comprehensive interventions, including regular doses of non-sedating H1-antihistamine and acupuncture intervention, in outpatient clinics affiliated with Guang’anmen Hospital for 6 weeks.

### Clinical Outcome Assessment

The clinical symptoms of all patients were assessed using the UAS7 3 days before the fMRI scan.

### Magnetic Resonance Imaging Data Acquisition

Patients discontinued antihistamine drugs and interventions 3 days prior to the scan. During the scan, the patient did not have any itching. The functional MRI scans were conducted on a 3.0 T Siemens MAGNETOM Skyra MRI system equipped with a standard 20-channel head coil. T2 WI data was first acquired to exclude any lesions and abnormalities. T1-weighted high-resolution structural images were acquired with the three-dimensional fast spoiled gradient-echo sequence (TR 5000 ms, TE 2.98 ms, matrix 256 × 256, FOV 256 × 240 mm, FA 1 = 4°, slice thickness 1 mm, gap 0 mm, 176 slices). Blood oxygen level-dependent (BOLD) fMRI images encompassing the whole brain were collected with the gradient echo EPI sequence (TR 2500 ms, TE 30 ms, matrix 70 × 70, FOV 210 × 210 mm, FA = 90°, slice thickness 3 mm, gap 0 mm, 43 slices, paralleled by AC-PC line). During the 369 s (dummy scan for the first 9 s) resting state fMRI scan, subjects were asked to lie still with their eyes closed.

### Data Preprocessing and Calculation of ReHo

Preprocessing was performed using DPARSF software of Dpabi V2.3 (a toolbox for Data Processing and Analysis of brain imaging; http://rfmri.org/dpabi) [18], which is based on SPM12 (http://www.fil.ion.ucl.ac.kr/spm) and a Resting-state fMRI Data Analysis Toolkit (http://www.restfmri.net) [19] in MATLAB (The MathWorks, Natick, MA, USA). The specific steps are as follows: (1) checked the scanning image quality for each participant and transformed EPI DICOM to NIFTI; (2) removed first four time points; (3) functional slice-timing corrected; (4) spatially realigned; (5) segmented the structural image into gray matter, white matter, and cerebrospinal fluid (CSF); (6) removed the Friston 24 head motion parameters and CSF signals as regressors; (7) normalized the images using standard Montreal Neurological Institute (MNI) templates with a resolution of 3 × 3 × 3 mm; (8) subjects with head movements exceeding 2 mm on any axis or with head rotation greater than 2° were excluded; (9) detrended data; and (10) filtered the data using the low frequency band (0.01–0.08 Hz).

ReHo values were calculated using DPARSF. Individual ReHo maps, measuring the correlation of the time series between a given voxel and its 26 nearest neighbors, were generated by calculating the Kendall coefficient of concordance (KCC) [15]. For standardization purposes, each ReHo map was divided by the global mean ReHo of each participant. Then, standard normal Z transformation was performed for each ReHo to generate the zReHo map. Finally, the zReHo maps were smoothed with a Gaussian kernel of 8-mm full-width at half maximum (FWHM). Ultimately, we obtained each participant’s szReHo map for the following statistical analysis.

### Seed-Based Resting State Functional Connectivity Analysis

To further explore the circuits based on brain regions that showed significant differences in the above analysis, we also performed seed-based resting state functional connectivity analyses. We used the CONN-fMRI Functional Connectivity Toolbox v17.a [20] to perform the seed-based resting state functional connectivity. The preprocessing are as follows: (1) functionally realigned and unwarped, (2) functionally centered to coordinates, (3) functional slice-timing corrected, (4) structural center to coordinates, (5) structural segmentation and normalization, (6) functional normalization, (7) functional outlier detection (ART-based identification of outlier scans for scrubbing), and (8) functional smoothing with an 8-mm FWHM. Band-pass filtering was performed using the low frequency band (0.01–0.08 Hz).

Two seeds were chosen for the seed-based resting state functional connectivity analysis [21]: (1) generated on the basis of the peak point of ReHo in the cerebellum (− 12, − 72, − 18, 8 mm) and (2) the cluster of SI/MI/SMA obtained from ReHo analysis (comparison of pre vs post intervention). The rs-FC of the seed with the whole-brain was compared between CSU (*n* = 40) patients and HCs (*n* = 40), as well as between the CSU patients (*n* = 32) before and after intervention. Both seeds were produced by WFU-Pick Atlas software. Functional connectivity measures were computed between a seed and every other voxel in the brain. The residual BOLD time course was extracted from a given seed, and then first-level correlation maps were estimated by computing Pearson’s correlation coefficients between that time course and the time courses of all other voxels in the brain. Correlation coefficients were transformed into Fisher’s *Z*-scores to increase normality and allow for improved second-level general linear model analyses.

### Statistical Analysis

#### Behavioral Data

Statistical analyses were performed using SPSS 18.0 (SPSS Inc., Chicago, IL). The mean ± SD was used for normally distributed continuous variables. UAS7 before and after intervention were compared using a paired-sample *t* test.

#### Imaging Data

SPM12 was used to examine group differences in ReHo and seed-based resting state functional connectivity. Two-sample *t* tests were performed between HCs and CSU patients before intervention. A paired *t* test was performed between patients before and after intervention. A threshold of voxel-wise *p* < 0.005 uncorrected and cluster-level *p* < 0.05 FWE corrected was applied for all fMRI data analysis.

The brain regions that demonstrated significant differences were identified as regions of interest (ROIs). The mean ReHo values or mean fisher *Z* values within the ROIs were then extracted for correlation analysis to explore their association with the clinical outcome.

## Results

### Clinical Characteristics

Forty patients (32 female and 8 male) and 40 healthy individuals were matched by age and gender. Thirty-two patients completed the intervention and were scanned at week 0 and week 6. Following a comprehensive intervention, UAS7 scores were significantly reduced (*p* < 0.001) (average ± SD: UAS7_pre_ = 30.2 ± 6.1; UAS7_post_ = 19.5 ± 7.3) (Table 1).

**Table 1:** Demographics and clinical characteristics of HC and CSU groups. Values are presented as mean ± SD

	HC (*n* = 40)	CSU group (n = 40)	CSU group completed the intervention (*n* = 32)	
Gender (male/female)	8/32	8/32	6/26	
Age (years)	42.6 ± 10.8	42.6 ± 10.8	43.6 ± 11.6	
Duration of illness (months)	–	103 ± 150	109 ± 158	
UAS7	–	30.8 ± 6.2	30.2 ± 6.1 (pre)	19.5 ± 7.3 (post)

### Regional Homogeneity Analysis Results

#### Comparison Between CSU Patients and HCs

**Fig. 1:**
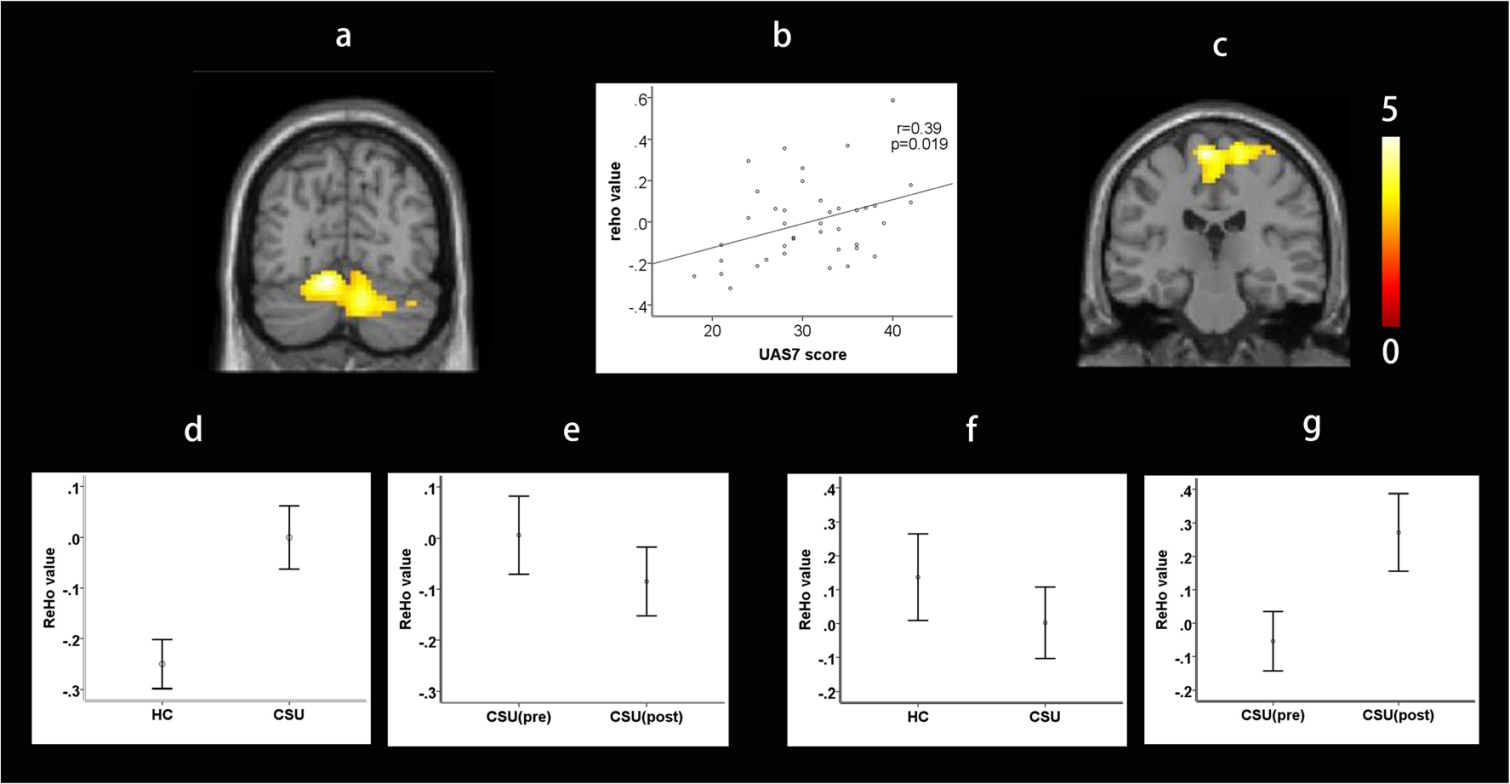
**a** ReHo values of the cerebellar cluster in CSU patients were significantly increased compared to HCs. **b** ReHo values of the cerebellar cluster in CSU patients at baseline were positively correlated with the UAS7. **c** After a comprehensive intervention, ReHo values were significantly increased at SI/MI/SMA. **d** ReHo values of the cerebellar cluster in the CSU group were higher than HCs. **e** Following an intervention, ReHo values of the cerebellar cluster revealed a significant decrease. **f** ReHo values of the cluster, including, SI, MI, and SMA, in the CSU group were lower than the HC group, but the differences were not significant (*p* = 0.10). **g** Following an intervention, the ReHo values of the cluster including SI, MI, and SMA were significantly increased

Compared to healthy controls, CSU patients showed significantly increased ReHo values in the cerebellum. No other significant regions were identified (Table 2; Fig. 1a, d).

**Table 2:** Brain regions with significant differences in ReHo values between CSU patients and HCs

Contrast	Brain regions	MINI coordinates			Peak *Z*-value	Number of voxels in the cluster
		*X*	*Y*	*Z*		
CSU > HC	Bilateral cerebellum	− 12	− 72	− 18	4.57	904
HC > CSU	None					
Post > pre	Bilateral SI/MI/SMA	− 6	− 24	69	4.15	507
Post < pre	None

To further test the association between the ReHo values and UAS7, we extracted the ReHo values (*n* = 40) from the cerebellar cluster and performed a correlation analysis. The results showed that the ReHo values at baseline were positively associated with UAS7 (*r* = 0.39, *p* = 0.019) (Fig. 1b).

#### Comparison of Pre vs Post Intervention in CSU Patients

Following a comprehensive intervention, a paired *t* test revealed that patients’ ReHo values (*n* = 32) significantly increased in the sensorimotor region, including the bilateral primary somatosensory cortex (SI), primary motor cortex (MI), and supplementary motor area (SMA) (Table 2; Fig. 1c, g). ReHo values extracted from the sensorimotor cluster were subjected to analysis. The ReHo values of CSU patients were lower than those of HCs (*n* = 40), but there were no significant differences (*p* = 0.10) (Fig. 1f).

Although we did not find significant pre vs post intervention differences in the cerebellum of CSU patients during whole brain analysis at the threshold we set, a paired *t* test (*n* = 32) showed a significant decrease in extracted average ReHo values of the cerebellar cluster after a comprehensive intervention (*p* = 0.05) (Fig. 1e).

### Seed-Based Resting-State Functional Connectivity Analysis Results

To further explore the circuits based on brain regions that showed significant differences in the above analysis, we also performed seed-based resting state functional connectivity analyses using two seeds derived from ReHo analysis: cerebellum and SI/MI/SMA. The rs-FC of the seed with the whole-brain was compared between CSU (*n* = 40) patients and HCs (*n* = 40), as well as between the CSU patients (*n* = 32) before and after intervention.

#### Rs-FC Results Using a Cerebellar Seed

##### CSU Vs HCs

CSU patients demonstrated increased rs-FC relative to HCs at the bilateral anterior cingulate cortex, medial prefrontal cortex, right ventral striatum, angular gyrus, supramarginal gyrus, temporal gyrus, orbitofrontal cortex, and dorsal lateral prefrontal cortex (Table 3). There were no significant decreases observed at the threshold we set.

**Table 3:** Regions showing significant rs-FC differences with the seeds

Seed	Contrast	Brain regions	Cluster size (voxels)	Peak *Z*-score	MNI coordinates		
					*X*	*Y*	*Z*
Cerebellum	CSU > HC	Bilateral anterior cingulate cortex, medial prefrontal cortex, right ventral striatum	1482	4.15	10	68	4
		R angular gyrus, supramarginal gyrus	820	4.04	46	− 50	36
		R temporal gyrus	787	4.02	60	2	− 18
		R orbitofrontal cortex	456	3.63	34	46	6
		R dorsal lateral prefrontal cortex	331	3.38	22	46	42
	HC > CSU	None-					
	Post < pre	R middle frontal gyrus, precentral gyrus, postcentral gyrus	871	4.85	40	2	58
		R orbitofrontal cortex	556	3.80	42	42	− 4
	Post > pre	None					
SI/MI/SMA	Post < pre	R middle and superior temporal gyrus	720	4.26	50	− 50	12
		L dorsal lateral prefrontal cortex	705	4.25	− 26	50	42
		bilateral middle cingulate cortex	1127	3.95	0	8	26
		L putamen, caudate, accumbens, thalamus		3.87	− 26	− 2	2
	HC > CSU	None					
	CSU > HC	None					
	Post > Pre	None					

##### Pre vs Post

**Fig. 2:**
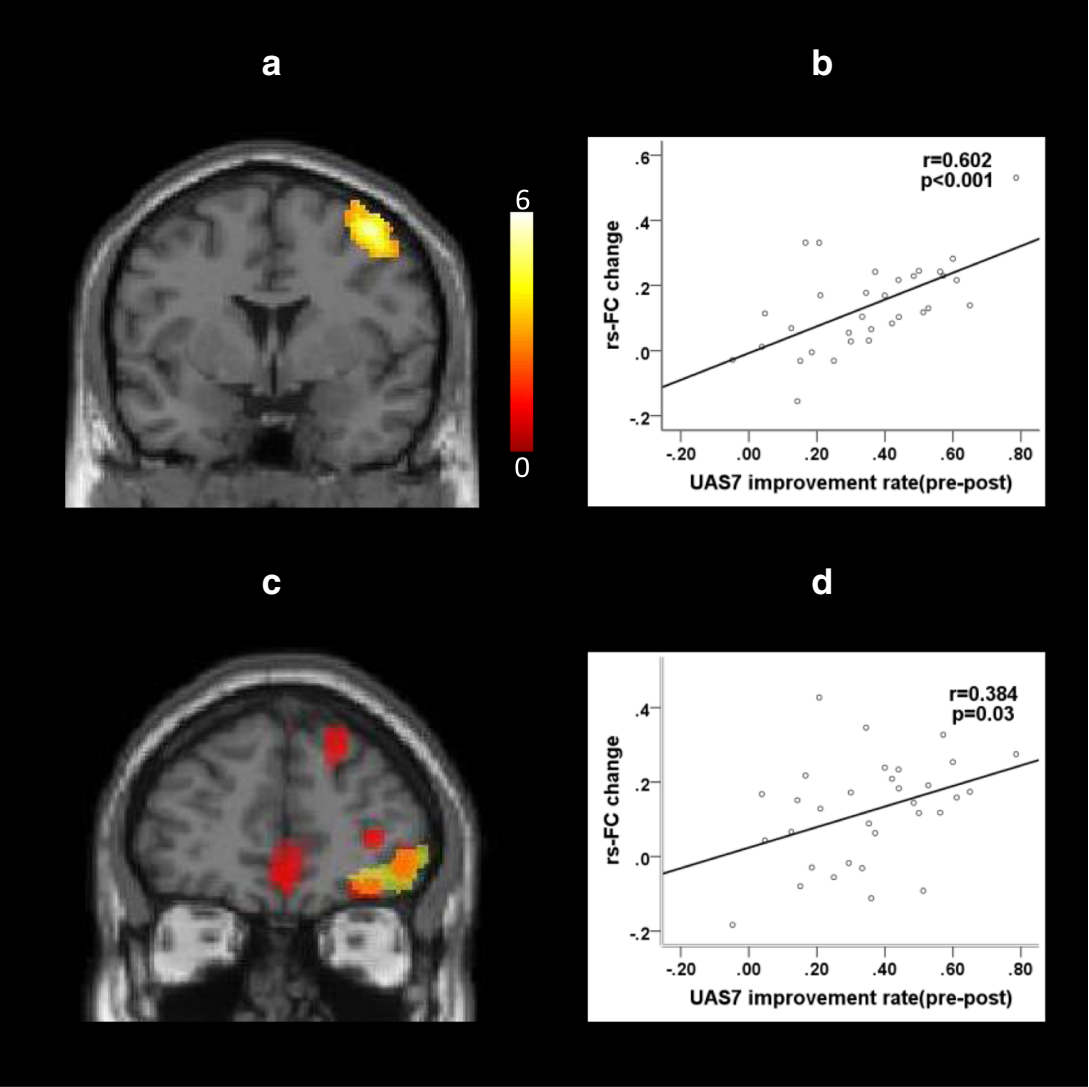
Resting state functional connectivity results. **a** After a comprehensive intervention, CSU patients showed decreased rs-FC between the cerebellum and the right middle frontal gyrus/precentral gyrus/postcentral gyrus (pre > post, *Y* = 2). **b** Correlation between clinical improvement, as indicated by UAS7, and rs-FC changes between the cerebellum and the right middle frontal gyrus/precentral gyrus/postcentral gyrus (*r* = 0.60, *p* < 0.001). **c** Two comparisons (CSU patients vs HCs and pre vs post intervention in CSU patients) overlap at the right orbitofrontal cortex (cerebellar seed, *Y* = 46; yellow areas: pre > post; red areas: CSU > HCs). **d** Correlation between clinical improvements, as indicated by UAS7, and rs-FC changes between the cerebellum and right orbitofrontal cortex (*r* = 0.38, *p* = 0.03)

After a comprehensive intervention, CSU patients showed decreased rs-FC at the right middle frontal gyrus/precentral gyrus/postcentral gyrus and dorsal orbitofrontal cortex (Table 3; Fig. 2a, c). Interestingly, we found two comparisons (CSU patients vs HCs and pre vs post intervention in CSU patients) overlapped at the right orbitofrontal cortex (Table 3; Fig. 2c). The improvement rate values, as measured by UAS7, were significantly positively correlated with reduced functional connectivity values in the right middle frontal gyrus/precentral gyrus/postcentral gyrus (*r* = 0.60, *p* < 0.001) and right orbitofrontal cortex (*r* = 0.38, *p* < 0.05) (Fig. 2b, d).

#### Rs-FC Results Using the Cluster (SI/MI/SMA) as ROIs

##### Pre vs Post

Following a comprehensive intervention, the rs-FC decreased at the bilateral middle cingulate cortex, right middle and superior temporal gyrus, left dorsal lateral prefrontal cortex, putamen, caudate, accumbens, and thalamus (Table 3).

## Discussion

In this study, we investigated rs-FC changes in CSU patients as compared to healthy controls (HCs) and the modulation effect of a comprehensive intervention. We found that the ReHo values at the cerebellum were significantly increased in CSU patients when compared with HCs. Following the intervention, ReHo values at the cerebellum were significantly decreased, while ReHo values at the bilateral SI/MI/SMA were significantly increased. The seed based rs-FC analysis using the regions with significant ReHo changes showed (1) significant rs-FC changes between the cerebellum and reward networks at the ventral striatum, ACC, and prefrontal cortex compared with HCs, and showed significant rs-FC changes between the cerebellum and sensorimotor areas in CSU patients pre vs post intervention and (2) significant rs-FC changes between SI/MI/SMA and reward networks including the putamen, caudate, accumbens, and thalamus after treatments.

The most common clinical manifestation of CSU is repeated itchy wheals and scratching. Scratching an itch, a pleasurable experience, is correlated with the intensity of the itch [22, 23]. This pleasurable experience is associated with the activation of reward regions [24, 25], even when there is no scratching behavior [26, 27]. Previous studies have suggested that both sensorimotor-related regions and reward regions are involved in the itch-scratch cycle [6–9]. Sensorimotor related regions, including primary somatosensory/motor area and cerebellum, not only involve cutaneous and muscle tissue [28–35] but also interact with brain regions involved in reward such as the putamen, caudate, and accumbens [36–41].

In this study, we found that in CSU patients, ReHo values at the cerebellum were significantly increased and that effective treatment could normalize (decrease) these alterations. In addition, we also found that the rs-FC between the cerebellum and brain regions associated with reward and sensorimotor processing were significantly altered, and these results are consistent with previous neuroanatomy studies, as well as a recent study from our group using the same data set. We found that compared with controls, CSU patients exhibited higher amplitude of low frequency fluctuations (ALFF) values in the right ventral striatum/putamen and increased gray matter volume in the right ventral striatum and putamen [42].

Neuroanatomy studies showed that the cerebellum sends projections to the cortical sensorimotor areas and reward regions via the thalamus [43–45]. At the same time, the cerebellum receives cortical input from the reward regions through the pontine nuclei and cortical input from sensorimotor regions through both the pontine nuclei and the inferior olive [44, 46]. Thus, we speculate that the cerebellum, SI/MI/SMA, and reward regions form a loop to process information in the itch-scratch cycle. The pathway from the cerebellum to the reward and sensorimotor regions by the cerebellar efferent nerve may be involved in the neuropathology of CSU. In addition, the pathways are composed of loops among the cerebellum, reward regions, and sensorimotor regions such as the loops between the reward regions with the sensorimotor regions via striato-thalamo-cortical circuitry [47]. Consistent with this study, following a comprehensive intervention, CSU patients exhibited decreased FC between the sensorimotor seed and both the striatum and the thalamus.

Based on the hypothesis of “control of mental and motor activities by internal models in the cerebellum” [48–52], cortico-cerebellar projections convey efferent copies of information from cortical areas to modular cerebellar internal models (neural representation of the external world [53]) so that they can efficiently mimic the information processing in the targets of those cortical areas [48, 53]. Through a learning process in the cerebellum, neural representations can simulate natural processes [48, 54]. This internal model is formed and adjusted as a movement is repeated [55]. Ultimately, the internal model in the cerebellum helps the brain to perform the movement precisely, without needing to refer to feedback from the moving body part [53]. In addition, the cerebellum might also encode internal models that reproduce the essential properties of mental representations in the cerebral cortex [53].

Specifically, “through learning” in the cerebellum, the cerebellum-reward-sensorimotor cortex pathway may form a loop to process the information of scratching the itch. Also, we speculate that when scratching the itch becomes an automatic process, the role of the sensorimotor cortex will be significantly attenuated, and the role of the cerebellum will become predominant. This is consistent with our finding that the cerebellum is the only region showing significant differences in CSU. Interestingly, following a comprehensive intervention, the ReHo values of the cerebellum significantly decreased, while the SI/MI/SMA significantly increased. These results further endorsed the important role of the cerebellum in the neuropathology of CSU and are partly consistent with previous studies showing that the cerebellum forms circuits with specific regions of the cerebral cortex to provide anatomical substrates for cerebellar involvement in sensorimotor and advanced cognitive processes [56–58].

In this study, we also found that the improvement rate values, as measured by the UAS7, were significantly positively correlated with reduced functional connectivity values between the cerebellum and the right precentral gyrus/postcentral gyrus and the right orbitofrontal cortex. The orbitofrontal cortex is an important brain region in the processing of reward [59]. The precentral gyrus is thought to encode motor actions [60], while the postcentral gyrus is thought to encode somatosensory information [61]. Both the precentral gyrus/postcentral gyrus and the orbitofrontal cortex are key brain regions in the processing of itch [6–10, 62]. The rs-FC changes between the cerebellum and precentral gyrus/postcentral gyrus and orbitofrontal cortex seem more sensitive to clinical improvement. These results indicate that multiple systems, including the reward and sensorimotor network, are both involved in the modulation of the itch-scratch cycle, and all these regions seem to interact with the cerebellum.

Accumulating evidence suggests that the skin and the brain are anatomically connected and functionally interacted in a bi-directional manner (the skin-brain axis and brain-skin axis) [63, 64]. For instance, the brain shares numerous mediators with skin through the hypothalamic-pituitary-adrenal axis (HPA axis) [63]. Studies have shown that the HPA axis may be altered in stress-related skin diseases, resulting in the activation of mast cells [65, 66], which are the primary effector cells in CSU [67]. The skin-brain connection can be complicated by considering that the skin has its own neuroendocrine system presumably as a result of its common origin with the brain [64]. At the same time, the brain may profoundly influence skin sensations through emotional and cognitive aspects [64]. In addition, a brain-derived nerve growth factor can mediate or enhance skin inflammation [66, 68]. Thus, the alternation of cerebellum regional coherence and cerebellum-reward-sensorimotor loops detected in CSU may represent the etiology of CSU. The normalization of functional connectivity after intervention accompanied with symptom relief suggests that a comprehensive and effective intervention may achieve clinical improvement by regulating brain plasticity.

We applied ReHo in this study. As a data-driven local functional connectivity data analysis method, ReHo can inform us of the key brain regions rather than the networks derived from other functional connectivity methods such as independent component analysis. It provides a new angle to analyze resting state functional connectivity data. However, ReHo has its disadvantages. First, it may be sensitive to spatial smoothing. In addition, it may be confounded by fluctuations of non-neuronal area. Finally, its sensitivity may be influenced by the shape of brain regions [69].

## Conclusions

**Fig. 3:**
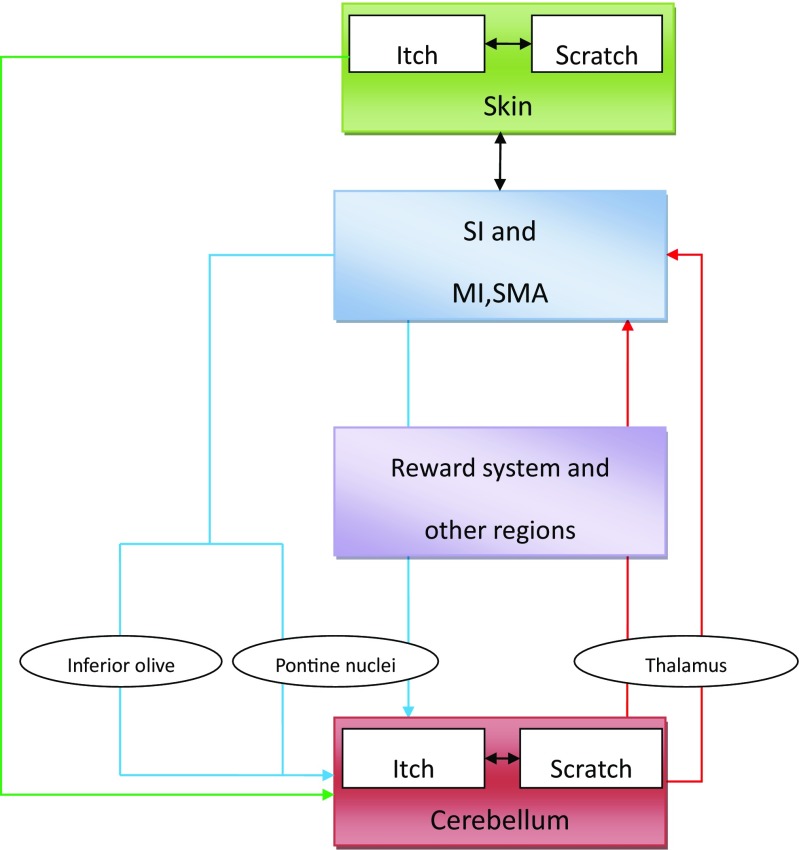
Itch-scratch cycle model in the central nervous system. The sensorimotor regions, such as SI, MI, SMA, and cerebellum, play an important role in processing itching signal. All the above signals may be learned, re-encoded, and memorized in the cerebellum, which may be modified by the reward system. In CSU, the sensorimotor-reward-cerebellum pathway (blue line) may be responsible for projecting itch-scratch signals to the cerebellum through cerebellar afferent nerve. And then, “through learning” in the cerebellum, the cerebellum-reward-sensorimotor pathway (red line) may be responsible for projecting imitative signals to the sensorimotor and reward regions through the cerebellar efferent nerve and play a key role in the neuropathology of chronic itch. Green line: the sensory signal from skin to the cerebellum

This study identifies the dysfunction of the cerebellum and the cerebellum-reward-sensorimotor loops in CSU. In addition, we also found that the SI/MI is more sensitive to clinical outcome changes, although there are no significant differences between the CSU patients and healthy controls at this area. Our results may provide valuable insights relevant to the neuropathology and the development of CSU (Fig. 3).

## Abbreviations

CSU: Chronic spontaneous urticaria
UAS7: Urticaria activity scores over 7 days
ReHo: Regional homogeneity
rs-FC: Resting-state functional connectivity
SI: Primary somatosensory cortex
MI: Primary motor cortex
SMA: Supplementary motor area
ROIs: Regions of interest
